# Salinity Mediates *Tamarix chinensis* Litter Decomposition to Enhance Soil Enzyme Activity in Coastal Saline–Alkali Soils

**DOI:** 10.3390/plants14172674

**Published:** 2025-08-27

**Authors:** Yue Lu, Lingtai Kong, Shihui Li, Pan Lun, Fanglei Gao, Qiqi Cao, Jiangbao Xia

**Affiliations:** 1 Shandong Key Laboratory of Eco-Environmental Science for the Yellow River Delta, Shandong University of Aeronautics, Binzhou 256603, China; yue02152023@163.com (Y.L.); lishihui1024@163.com (S.L.); bzhmgfl@163.com (F.G.); qiqicao163@163.com (Q.C.); 2 Binzhou Hydrographic Bureau, Binzhou 256609, China; konglingtai@163.com (L.K.); lunpan1234@163.com (P.L.)

**Keywords:** litter, saline–alkali soil, soil salinity, soil nutrients, soil improvement, *Tamarix chinensis*

## Abstract

The aim of this study was to explore the decomposition characteristics of *Tamarix chinensis* litter and its soil-improving capacity under different salinities. Four treatments were designed: a control (CK) treatment without saline water injection and three treatments encompassing slightly (SS, 0.4% soil salinity), moderately (SM, 0.8%), and highly saline (SH, 1.2%) conditions. *T. chinensis* litter at three degrees of decomposition (undecomposed, semidecomposed, and already decomposed) was studied. After 180 days, the litter substrate quality, 0–10 cm soil physicochemical properties, and enzyme activities were measured. Correlation analysis and structural equation modeling were employed to elucidate the interactions and response patterns among soil salinity, the decomposition characteristics of *T. chinensis* litter, and the physicochemical properties and enzyme activities of surface soil. The results revealed the following: (1) With increasing soil salinity, the contents of litter lignin, cellulose, total carbon and nitrogen residues first decreased but then increased, reaching minima under SM, whereas the content of hemicellulose residue exhibited the opposite trend. With increasing degree of litter decomposition, the contents of lignin and cellulose residues decreased, whereas the contents of hemicellulose, total nitrogen and phosphorus residues increased. (2) With increasing soil salinity, the soil water content, organic matter content, total nitrogen content, and activity of several enzymes increased, peaking under SH. The pH performance followed the order of SS > SM > CK > SH. The total carbon and phosphorus contents first increased but then decreased, with a maximum under SS. The activity of N-acetylamino glucosidase first decreased but then increased and was greatest at moderate and high salinities. (3) The soil water content and level of enzyme activity were significantly correlated with the litter substrate quality. Salinity negatively affected litter substrate residues but positively affected soil physicochemical properties. Litter decomposition under different soil salinities indirectly influenced soil enzymes by affecting soil properties, whereas salinity modulated soil properties directly or through litter decomposition. *T. chinensis* litter decomposition notably increased enzyme activity in moderate- to high-salinity alkali coastal soils, offering insights for low-efficiency *T. chinensis* forest management and saline–alkali soil remediation in the Yellow River Delta.

## 1. Introduction

The nutrients returned to soil through the leaching and decomposition of litter are important for the development of plant roots and play important roles in shaping material circulation, energy transfer, and the maintenance of ecosystem function and structure [1]. Litter decomposition can improve the granular structure and increase the water permeability and water retention of soil. Soil organic matter and mineral nutrients become enriched, which increases soil fertility. This promotes microbial activity and contributes to the formation of a favorable soil environment [2,3]. Litter significantly influences the structure and physicochemical properties of saline–alkali soils. Humus produced by litter decomposition can increase soil microbial activity, which can improve the structure and increase the permeability and water retention capacity of saline–alkali soil and thus accelerate salt leaching [4,5,6].

Soil salinization is a worldwide problem in various ecosystems. The area of saline–alkali soil in China is extensive, and saline–alkali soils are classified as slightly, moderately and highly saline–alkali soils [7,8]. Coastal saline–alkali soil typically results in inefficient ecosystem functions, and coastal saline–alkali soils are typically distributed in the Yellow River Delta of China. This area is subject to the dual influences of land and ocean, and the degree of soil salinity differs notably across this area [9,10]. *Tamarix chinensis* is the most widely distributed shrub plant in the Yellow River Delta region, and it is also an important plant in the Southern Red and Northern Willow coastal wetland ecological restoration project, playing important roles in driving the ecological improvement and ecological restoration of coastal saline–alkali soil [5]. Recent research on *T. chinensis* has focused mainly on its physiological and ecological characteristics [11,12], as well as population dynamics, distribution characteristics, and ecological adaptability [10]. Research on litter has focused largely on the decomposition characteristics of litter and the corresponding influencing factors in mangrove forests [1,13]. In a study of the effects of climate change on litter decomposition from Central Europe to Northern Europe, macroclimatic conditions were found to affect the decomposition of specific types of litter but did not affect overall litter decomposition or nitrogen release [14]. Research on *T. chinensis* litter has focused mainly on the effects of extreme conditions such as drought, sand burial and salt stress on the decomposition rate of *T. chinensis* litter in the Tarim desert ecosystem [15,16]. Yuan et al. reported that sand burial significantly positively influenced litter decomposition and nutrient release in extremely arid areas [15]. In the Yellow River Delta region, the effects of *T. chinensis* litter input on soil organic carbon (SOC) components and soil quality have been investigated. Li et al. reported that the addition of *T. chinensis* litter effectively reduced the soil density and salt content and increased soil nutrient levels and organic carbon storage in saline–alkali areas [17]. However, research on the decomposition characteristics of *T. chinensis* litter in coastal saline–alkali soils is limited. Research on litter under saline soil conditions has focused primarily on the effects of brackish water irrigation and secondary salinization on litter decomposition and soil organic carbon mineralization, and relevant studies on the characteristics of litter decomposition in *Suaeda salsa* [3], *Phragmites australis* [6], and *Spartina alterniflora* [6] areas in the Yellow River Delta have been carried out. However, research on the decomposition characteristics of *T. chinensis* litter and its effects on the physicochemical properties and enzyme activities of surface soil at different soil salinities is limited, which limits the management of inefficient *T. chinensis* forests. Notably, management strategies for improving soil quality in the litter layer are lacking. Therefore, research on the interactions and relationships among soil salinity, the decomposition characteristics of *T. chinensis* litter, and the physicochemical properties and enzyme activities of surface soils in coastal saline–alkali soils in the Yellow River Delta is urgently needed.

Therefore, in this study, slightly saline (SS), moderately saline (SM) and highly saline (SH) soils with salinities of 0.4%, 0.8% and 1.2%, respectively, were simulated, and soil without saltwater injection was employed as the control (CK). *T. chinensis* litter with different degrees of decomposition was investigated under different soil salinities. By analyzing changes in the substrate quality of the litter and the physicochemical properties and enzyme activities of the surface soil, the decomposition characteristics of *T. chinensis* litter with different degrees of decomposition were explored under various soil salinities. The goal of this study was to clarify the patterns of influence on the physicochemical properties and enzyme activities of surface soils. The interactions among soil salinity, the decomposition characteristics of *T. chinensis* litter, and the physicochemical properties and enzyme activity of surface soil were revealed to provide a reference for managing low-efficiency *T. chinensis* forests in coastal saline–alkali soil and to determine ways to improve saline–alkali soil.

## 2. Results

### 2.1. Characteristics of the Variations in the Substrate Quality of T. chinensis Litter

**Figure 1:**
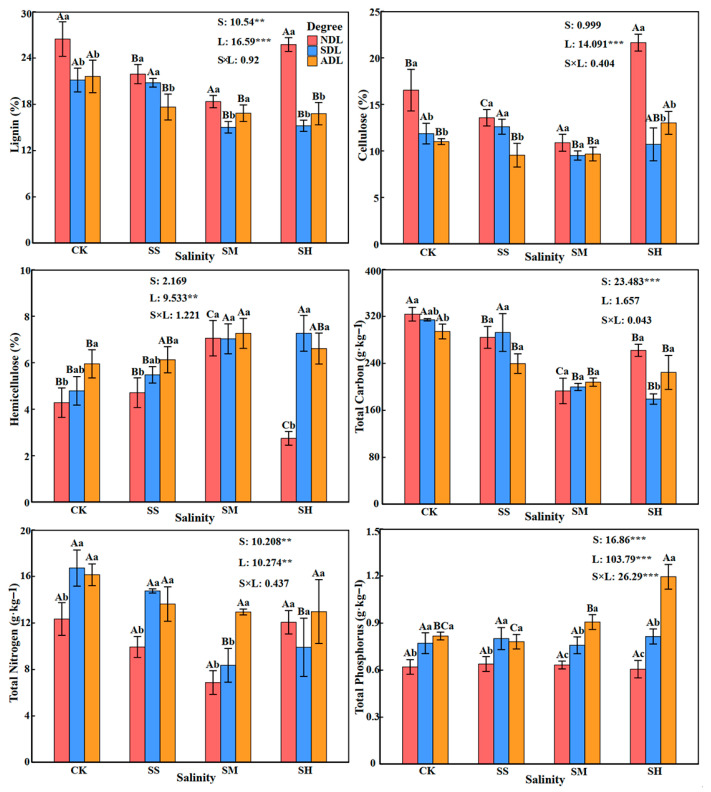
Substrate quality of *T. chinensis* litter under different salinity conditions. Note: The error bars in the figure represent the SDs. S: soil salinity content; L: degree of litter decomposition. *** and ** indicate that *p* is significantly different at the 0.001 and 0.01 levels, respectively. Different capital letters represent significant differences in various indicators between different soil salinities at the same degree of litter decomposition (*p* < 0.05), and different lowercase letters represent significant differences in various indicators between different degrees of litter decomposition at the same soil salinity (*p* < 0.05).

The substrate quality of *T. chinensis* litter was significantly (*p* < 0.05) or very significantly (*p* < 0.01) affected by the soil salinity, degree of litter decomposition or their interaction (Figure 1). There was a significant difference in the substrate quality of litter with the same degree of decomposition among different soil salinities (*p* < 0.05). With increasing soil salinity, the contents of lignin, cellulose, total carbon and total nitrogen residues in *T. chinensis* litter first decreased but then increased, and the residue contents were the lowest at moderate salinity; notably, the amounts released were the greatest, and they were 27.45%, 23.70%, 35.54% and 37.65% lower than those in the CK treatment, respectively. The content of hemicellulose residue first decreased but then increased and was the highest at moderate salinity; notably, the amount released was the lowest, which was 42.07% greater than that in the CK treatment. The total phosphorus residue content in the already decomposed litter (ADL) increased with increasing soil salinity, and the difference between the total phosphorus residue contents in the undecomposed litter (NDL) and semidecomposed litter (SDL) with increasing soil salinity was not significant (*p* > 0.05).

There were significant differences (*p* < 0.05) in the substrate quality of litter with different degrees of decomposition at the same soil salinity. The contents of lignin and cellulose residues in SDL and ADL were significantly lower than those in NDL (*p* < 0.05), and the values were 21.94% and 21.18% and 28.64% and 30.96% lower than those in NDL, respectively. In contrast, the trends in the contents of litter hemicellulose and total phosphorus residues varied along opposing directions, with the levels of these factors in SDL and ADL increasing by 30.70% and 38.09% and 25.94% and 48.12%, respectively, compared with those in NDL. Except at high salinity, the total nitrogen residue contents in SDL and ADL were significantly greater than those in NDL (*p* < 0.05), and the values were 20.69% and 35.13% greater than those in NDL, respectively. Compared with those in ADL, the substrate quality of litter residues in SDL were greater under the CK treatment and slightly saline conditions, and those in ADL exceeded those in SDL with increasing soil salinity.

### 2.2. Physicochemical Properties of Surface Soil

**Figure 2:**
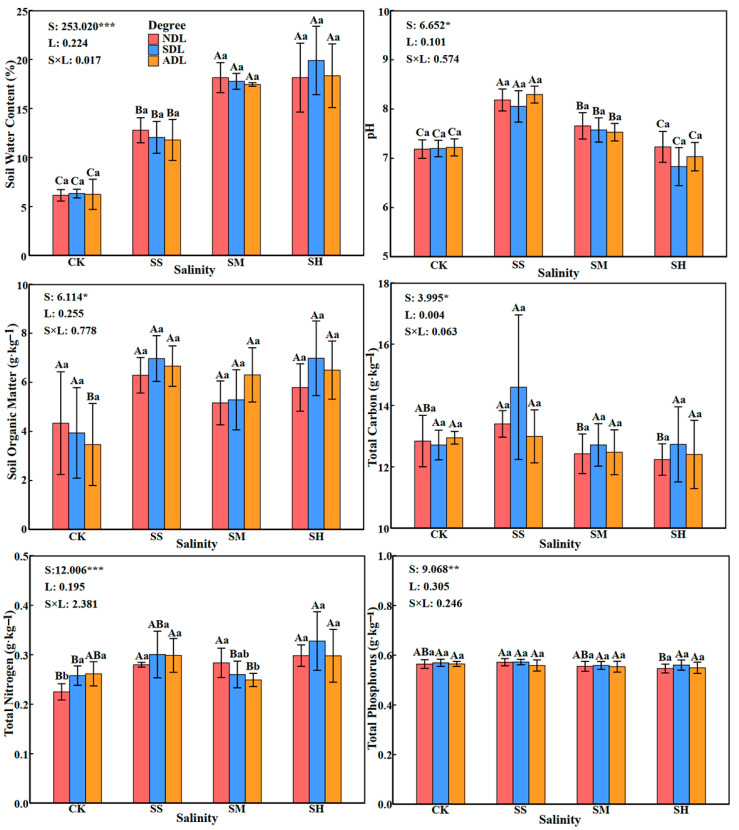
Physicochemical properties of surface soil under different salinity conditions. Note: The error bars in the figure represent the SDs. S: soil salinity content; L: degree of litter decomposition. ***, ** and * indicate that *p* is significantly different at the 0.001, 0.01, and 0.05 levels, respectively. Different capital letters represent significant differences in various indicators between different soil salinities at the same degree of litter decomposition (*p* < 0.05), and different lowercase letters represent significant differences in various indicators between different degrees of litter decomposition at the same soil salinity (*p* < 0.05).

The physicochemical properties of surface soil were significantly (*p* < 0.05) or very significantly (*p* < 0.01) affected by soil salinity but were not significantly affected by the degree of litter decomposition or the interaction between these terms (*p* > 0.05; Figure 2). The effects of litter with the same degree of decomposition on soil physicochemical properties significantly differed (*p* < 0.05) across different soil salinities. With increasing soil salinity, the soil water content increased significantly, and the water contents at slight, moderate and high salinities were significantly greater than those in the CK treatment (*p* < 0.05), with values that were 1.96, 2.85 and 3.01 times greater, respectively. The soil pH and total carbon and total phosphorus contents tended to increase and then decrease and reached a maximum at slight salinity. Additionally, compared with those in the CK treatment, the pH values in the treatments with slight and moderate salinities were significantly greater (*p* < 0.05). The contents of soil organic matter and total nitrogen in the treatments with slight, moderate and high salinities were greater than those in the CK treatment. The content of soil total nitrogen followed the order of SH > SS > SM > CK, and the values in the salinity treatments were 24.14%, 18.11% and 6.54% greater than those in the CK treatment, respectively.

There were significant differences (*p* < 0.05) in the effects of litter with different degrees of decomposition on the soil total nitrogen content at the same soil salinity and no significant differences (*p* > 0.05) in the effects on the remaining soil physicochemical properties. With increasing degree of litter decomposition, the soil total nitrogen content increased gradually in the CK and slight-salinity treatments, decreased gradually at moderate salinity, and tended to increase and then decrease at high salinity. The soil total nitrogen content of ADL was significantly lower than that of NDL at moderate salinity (*p* < 0.05) and was 12.17% lower than that of NDL.

### 2.3. Ecological Stoichiometric Analysis of T. chinensis Litter and Surface Soil

#### 2.3.1. Ecological Stoichiometric Ratios of C, N, and P in *T. chinensis* Litter

**Figure 3:**
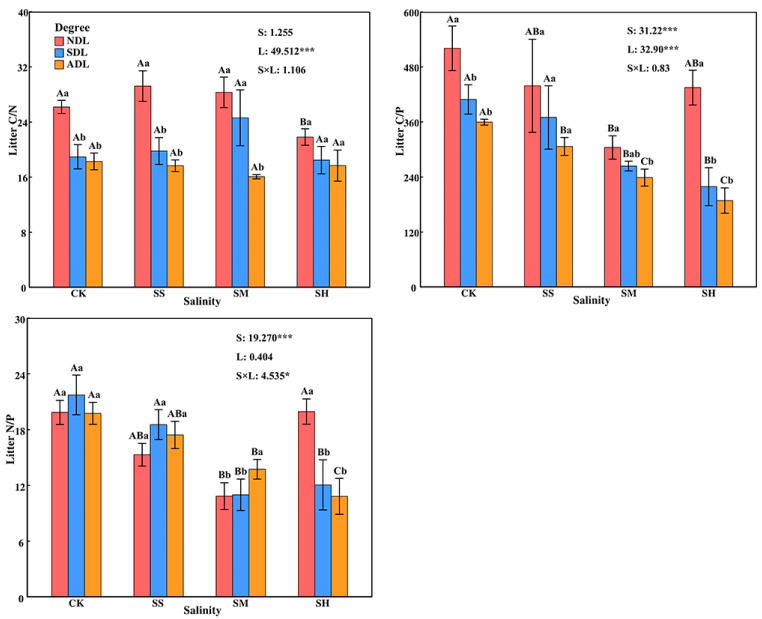
Ecological stoichiometric ratios of C, N, and P in *T. chinensis* litter under different salinity conditions. Note: The error bars in the figure represent the SDs. S: soil salinity content; L: degree of litter decomposition. *** and * indicate that *p* is significantly different at the 0.001 and 0.05 levels, respectively. Different capital letters represent significant differences in various indicators between different soil salinities at the same degree of litter decomposition (*p* < 0.05), and different lowercase letters represent significant differences in various indicators between different degrees of litter decomposition at the same soil salinity (*p* < 0.05).

The C:N:P stoichiometry of the *T. chinensis* litter was significantly (*p* < 0.05) or very significantly (*p* < 0.01) affected by the soil salinity, degree of litter decomposition or their interaction (Figure 3). The C:N:P stoichiometry of *T. chinensis* litter with the same degree of decomposition differed significantly (*p* < 0.05) across different soil salinities. With increasing soil salinity, the C/N of the litter first increased but then decreased and was the lowest under high salinity. The C/P and N/P of the litter first decreased but then increased, and overall, both ratios indicated the order of SM < SH < SS < CK, with the values in the various salinity treatments of 38.12%, 34.65%, 34.48% and 41.08%, 30.18%, 16.42% lower than those in the CK treatment, respectively.

There were significant differences (*p* < 0.05) in the C:N:P of litter stoichiometry with different degrees of decomposition at the same soil salinity. With increasing degree of litter decomposition, the litter C/N and C/P decreased significantly, and the ratios in ADL were significantly lower than those in NDL under moderate salinity (*p* < 0.05), by 43.26% and 21.54%, respectively; however, the N/P of the litter showed the opposite trend, with that in ADL being 26.60% higher than that in NDL. The C/P and N/P in SDL and ADL at high salinity were significantly lower (*p* < 0.05) than those in NDL, by 49.64%, 56.64% and 39.52%, 45.73%, respectively.

#### 2.3.2. Ecological Stoichiometric Ratios of C, N, and P in Surface Soil

**Figure 4:**
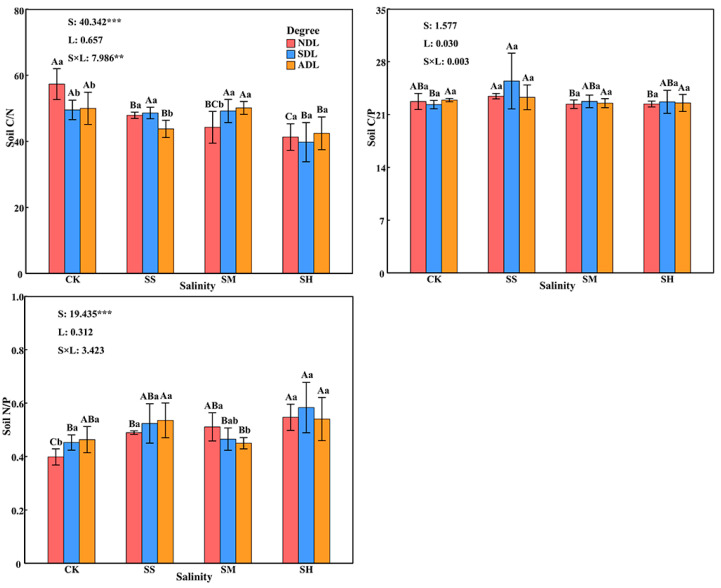
Ecological stoichiometric ratios of C, N, and P in the surface soil under different salinity conditions. Note: The error bars in the figure represent the SDs. S: soil salinity content; L: degree of litter decomposition. *** and ** indicate that *p* is significantly different at the 0.001 and 0.01 levels, respectively. Different capital letters represent significant differences in various indicators between different soil salinities at the same degree of litter decomposition (*p* < 0.05), and different lowercase letters represent significant differences in various indicators between different degrees of litter decomposition at the same soil salinity (*p* < 0.05).

The C/N and N/P of the surface soil were very significantly (*p* < 0.01) affected by soil salinity along and by the interaction between soil salinity and the degree of litter decomposition and were not significantly affected by the degree of litter decomposition alone (*p* > 0.05; Figure 4). The effects of litter with the same degree of decomposition on the soil C:N:P stoichiometry significantly differed (*p* < 0.05) across different soil salinities. With increasing soil salinity, the soil C/N generally tended to decrease in the order of SH < SS < SM < CK (*p* < 0.05), with values in the salinity treatments 21.25%, 10.57% and 8.45% lower than those in the CK treatment, respectively. The soil C/P generally tended to increase and then decrease, with the highest value occurring at slight salinity. The soil N/P followed the order of SH > SS > SM > CK, with values in the salinity treatments 27.06%, 17.81% and 8.49% greater than those in the CK treatment, respectively.

There were significant differences (*p* < 0.05) in the effects of litter with different degrees of decomposition on the soil C/N and N/P under the same soil salinity, but there was no significant difference (*p* > 0.05) in the effects on the soil C/P. With increasing degree of litter decomposition, the soil C:N:P stoichiometry did not change significantly. The soil C/N in SDL and ADL under moderate salinity were significantly greater (*p* < 0.05) than those in NDL by 11.14% and 13.24%, respectively. The soil C/N in ADL was significantly lower than those in NDL and SDL under slight salinity, by 8.58%. The soil N/P in ADL was significantly lower than that in NDL under moderate salinity, by 11.96%.

### 2.4. Changes in the Enzyme Activities in the Surface Soil

**Figure 5:**
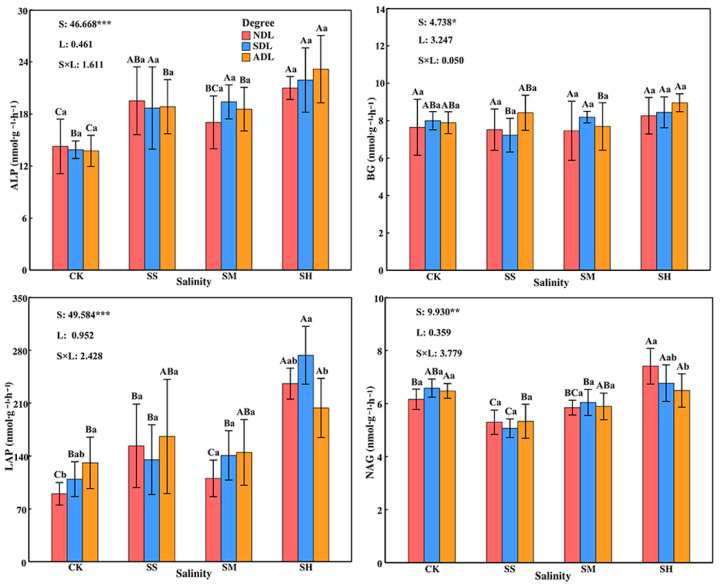
Enzyme activities in the surface soil under different salinity conditions. Note: The error bars in the figure represent the SDs. S: soil salinity content; L: degree of litter decomposition. ***, ** and * indicate that *p* is significantly different at the 0.001, 0.01, and 0.05 levels, respectively. Different capital letters represent significant differences in various indicators between different soil salinities at the same degree of litter decomposition (*p* < 0.05), and different lowercase letters represent significant differences in various indicators between different degrees of litter decomposition at the same soil salinity (*p* < 0.05).

The enzyme activities in the surface soil were significantly (*p* < 0.05) or very significantly (*p* < 0.01) affected by soil salinity and were not significantly affected by the degree of litter decomposition or the interaction between the two factors (*p* > 0.05; Figure 5). The effects of litter with the same degree of decomposition on soil enzyme activity differed significantly across different soil salinities (*p* < 0.05), and the activities of ALP, BG, LAP, and NAG under high salinity were greater than those in the CK and under slight and moderate salinities. The activity of ALP tended to decrease and then increase with increasing soil salinity, in the order of SH > SS > SM > CK (*p* < 0.05), with values in the salinity treatments 57.73%, 36.18% and 31.28% greater than those in the CK, respectively. The activity of BG was the lowest under slight salinity and was significantly lower under slight salinity than under moderate and high salinities in SDL (*p* < 0.05). The activity of LAP was significantly greater under high salinity than in the CK or under slight and moderate salinities (*p* < 0.05), with values 2.16 times greater. The activity of NAG was significantly lower under slight salinity than in the CK and under high salinity (*p* < 0.05), by18.31%.

There were significant differences (*p* > 0.05) in the effects of litter with different degrees of decomposition on the activities of LAP and NAG under the same soil salinity and no significant differences (*p* > 0.05) in the effects on the activities of ALP and BG. With increasing degree of litter decomposition, the activities of LAP and NAG increased in the CK and under moderate salinity, initially decreased and then increased under slight salinity, and either initially increased and then decreased or only decreased under high salinity. Under high salinity, the LAP activity in SDL was significantly greater than that in ADL (*p* < 0.05), and the NAG activity in ADL was significantly lower than that in NDL (*p* < 0.05) by 12.35%.

### 2.5. Relationships Between Indices of T. chinensis Litter Decomposition and Indices of Surface Soil Physicochemical and Enzyme Activities

#### 2.5.1. Correlation Analysis

**Figure 6:**
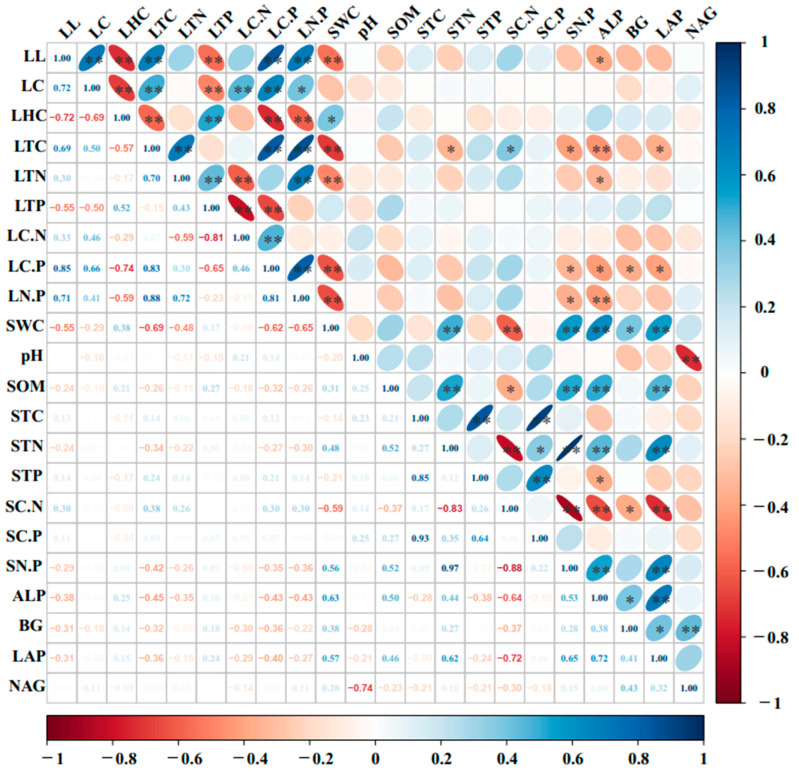
Correlation analysis between indices of *T. chinensis* litter decomposition and surface soil physicochemical properties and enzyme activities. Note: LL: lignin; LC: cellulose; LHC: hemicellulose; LTC: total litter carbon; LTN: total litter nitrogen; LTP: total litter phosphorus; LC.N: litter C/N; LC.P: litter C/P; LN.P: litter N/P; SWC: soil water content; SOM: soil organic matter; STC: soil total carbon; STN: soil total nitrogen; STP: soil total phosphorus; SC.N: soil C/N; SC.P: soil C/P; SN.P: soil N/P; ALP: alkaline phosphatase; BG: β-glucosyl enzyme; LAP: leucine aminopeptidase; NAG: N-acetylglucosaminidase. ** and * indicate that *p* is significantly relationship at the 0.01, and 0.05 levels, respectively.

Figure 6 shows that the water content of the surface soil was very significantly and negatively correlated with the residues of lignin and nutrients and the ecological stoichiometric ratios in the litter (r = −0.55, −0.69, −0.48, −0.62, and −0.65; *p* < 0.01). The surface soil total nitrogen content was significantly positively correlated with the soil water content (r = 0.48, *p* < 0.05). The activities of ALP and LAP were significantly (*p* < 0.05) or very significantly (*p* < 0.01) correlated with the residues of total carbon in the litter (r = −0.45, −0.36), the C/P of the litter (r = −0.43, −0.40) and the soil physicochemical properties. The activity of BG was negatively correlated with the ecological stoichiometric ratios of C, N, and P in the litter. There were no significant correlations (*p* > 0.05) among pH, soil organic matter content, total carbon content, total phosphorus content, C/P, NAG activity and substrate quality in the litter, but some correlations were detected among the soil factors.

#### 2.5.2. Analysis of the Structural Equation Modeling Results

**Figure 7:**
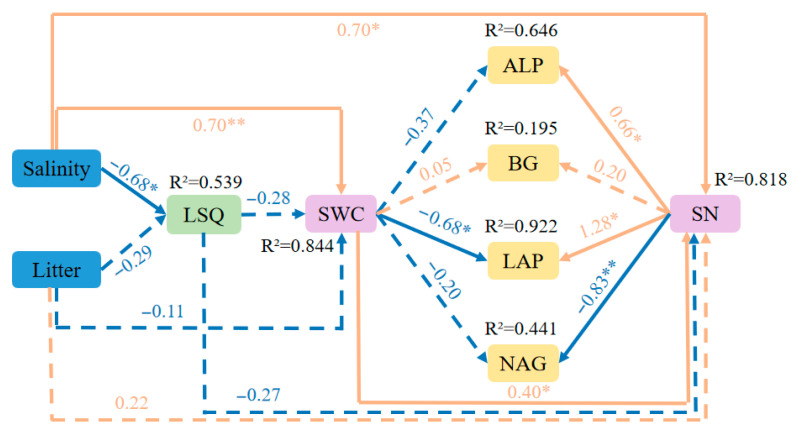
Structural equation model of indices of *T. chinensis* litter decomposition and soil environmental factors. Note: Salinity: soil salinity; Litter: degree of litter decomposition; LSQ: litter substrate quality residue; SWC: soil water content; ALP: alkaline phosphatase; BG: β-glucosidase; LAP: leucine aminopeptidase; NAG: N-acetylamino-glucosidase; SN: soil nutrient content. The number on the arrow is the standardized path coefficient. The solid line represents the relationship between variables (*p* < 0.05), and the dotted line represents the relationship between variables that are not significant (*p* > 0.05). Orange represents a positive effect, and blue represents a negative effect. ** and * indicate that *p* is significantly relationship at the 0.01, and 0.05 levels, respectively.

Structural equation modeling was used to analyze the direct or indirect effects of multiple variables on soil enzyme activities. The *p* > 0.05, GFI of 0.991, and CFI of 0.939 for the model indicated a reasonably high goodness of fit (Figure 7). SEM revealed that soil salinity had a significant effect on the residues of substrate quality in the litter, the soil water content and the soil nutrient levels (*p* < 0.05), and the degree of litter decomposition had no significant direct effect on those parameters. The soil water content and soil nutrient levels are directly or indirectly affected by soil salinity and litter substrate quality, thus affecting soil enzyme activities. Compared with the soil water content, the soil nutrient levels influenced the activities of ALP, BG, LAP and NAG more significantly. The soil water content significantly negatively influenced the activity of LAP (*p* < 0.05), with a path coefficient of −0.68. It significantly positively influenced soil nutrients (*p* < 0.05), with a path coefficient of 0.40, which in turn significantly influenced the activities of ALP, LAP and NAG (*p* < 0.05), with path coefficients of 0.66, 1.28 and −0.83, respectively.

## 3. Discussion

### 3.1. Characteristics of the Variation in the Substrate Quality of T. chinensis Litter

Lignin and cellulose are the primary components of litter, and the decomposition process results in the release of essential nutrients, such as carbon and nitrogen, which play significant roles in increasing the soil organic matter content [3,18], improving soil fertility and the water retention capacity [5], and increasing soil enzyme activity [18]. The ecological stoichiometric ratios of C, N, and P in the litter are critical indicators for characterizing the rate of litter decomposition. The lower the value is, the higher the decomposition rate [19]. The results of this study revealed that with increasing soil salinity, the residues of lignin, cellulose, total carbon and total nitrogen in *T. chinensis* litter first decreased and then increased. The lowest residual substrate quality of the litter, indicating the maximum nutrient release, was observed under moderate salinity. The C/P and N/P were generally in the order of SM < SH < SS < CK, and the decomposition rate exhibited a phenomenon of low inhibition and high promotion, peaking under moderate salinity, followed by high salinity. This phenomenon may be related to the soil water content. In this study, the soil water content was also greater under moderate- and high-salinity conditions and significantly greater than those under moderate-salinity conditions and in the CK. Moreover, the residues of lignin and nutrients in the litter, as well as the ecological stoichiometric ratios, were highly significantly negatively correlated with the surface soil water content (*p* < 0.01). This indirectly reflects the synergistic effect of increased soil water content and nutrient availability, which alleviated the inhibition of microbial activity caused by salinity stress to some extent and promoted leaching processes and decomposer activity, thereby increasing litter decomposition. These findings are consistent with previous research indicating that increased precipitation and soil moisture accelerate the decomposition of leaf litter from typical hygrophytic plants, such as *Carex thunbergii*, in the Shengjin Lake wetland [20]. A study of litter decomposition in different artificial *Robinia pseudoacacia* mixed forests in the Yellow River Delta also revealed that the litter decomposition rate was significantly positively correlated with the N and P contents and significantly negatively correlated with the C/N [21]. However, in mangrove ecosystems, the decomposition of *Kandelia obovata* and *Bruguiera gymnorrhiza* litter is most efficient at moderate salinity [13,22]. This discrepancy may be attributed to differences between the two types of ecosystems. The salt-tolerant microbial communities (e.g., *Halomonas*) in the Yellow River Delta may have competitive advantages at moderate and high salinities [23], whereas decomposers in mangrove ecosystems are more adapted to the low salinity fluctuations caused by periodic tides [24]. Additionally, the soil salinity composition in the Yellow River Delta is dominated by NaCl, whereas in mangrove areas, ions such as Mg^2+^ and Ca^2+^ are more abundant [10,25], which may inhibit the decomposition process by influencing enzyme activity.

### 3.2. Effects of T. chinensis Litter Decomposition on the Physicochemical Properties of the Surface Soil

An appropriate soil water content is important for improving the metabolic activity of microbes and the decomposition rate of organic matter, thereby promoting the recycling of soil nutrients [26]. The results of this study revealed that with increasing soil salinity, the soil water content increased significantly (*p* < 0.05) and was very significantly negatively correlated with the residues of lignin, nutrients, and ecological stoichiometric ratios in the litter (*p* < 0.01). This phenomenon may have occurred because the microbial activity was greater at moderate and high salinities, leading to an increase in the decomposition rate of *T. chinensis* litter. The humus produced during decomposition preserves surface soil moisture [5]. Soil pH primarily influences litter decomposition and nutrient release indirectly by affecting the structure of soil microbial communities and the efficiency of enzyme synthesis [27]. Soil organic matter serves as a vital nutrient source for both plants and soil microorganisms, reflecting soil fertility and quality [28]. In this study, the lowest pH was observed at high salinity, and it was negatively correlated with the soil total nitrogen content. Since the major source of soil total nitrogen relies on the decomposition of organic matter [29], there was a significant positive correlation between the two parameters (*p* < 0.01). With increasing soil salinity, the contents of soil organic matter and total nitrogen first decreased and then increased, reaching their highest levels at high salinity. These findings indicate that the decomposition of *T. chinensis* litter had the most obvious effect on reducing the soil pH under high-salinity conditions. This phenomenon may be attributed to increased microbial activity at high salinity, as highly active microorganisms release large amounts of organic acids during litter decomposition [29], thereby reducing the soil pH. Additionally, the acidic environment inhibits the activity of ammonia-oxidizing bacteria, reducing NH_3_ volatilization and promoting the retention of total nitrogen [30]. These findings are consistent with the observation that the addition of forest litter significantly reduces the pH of sticky yellow soil in southern China [31]. Another possible explanation is that the decomposition of *T. chinensis* litter increases nitrogen availability, alleviating the limitation of nitrogen in the soil. As a result, the content of soil total nitrogen gradually increases, leading to a reduction in soil pH. The interaction between these factors accelerates the rate of litter decomposition [30,32]. However, the contents of total carbon and total phosphorus in the soil exhibited opposite trends with respect to the contents of soil organic matter and total nitrogen. The soil C/N reached its lowest point at high salinity, whereas the N/P peaked. The lower the soil C/N is, the faster the mineralization or decomposition of organic matter in the soil [8]. The soil N/P reflects the decomposability of organic matter and serves as a diagnostic indicator of nitrogen saturation. A relatively high N/P indicates relative phosphorus deficiency in the soil [33]. Similarly, the soil C/P can reflect the mineralization capacity of phosphorus in the soil. The lower the C/P is, the greater the availability of phosphorus in the soil. In this study, there was no significant difference in the soil C/P (*p* > 0.05), but it was slightly lower at high salinity. This may have occurred because carbon and phosphorus are essential elements for microbial metabolism. Under moderate and high salinity, the total soil nitrogen content was relatively high, whereas the total phosphorus content was relatively low. Phosphorus is a limiting factor for microbial activity, and phosphorus in the soil is difficult to replenish effectively after consumption [8].

### 3.3. Effects of T. chinensis Litter Decomposition on Enzyme Activity in Surface Soil

Soil enzymes play critical roles in microbial metabolism and soil nutrient cycling. Their activity levels directly affect the turnover rate of soil organic matter and serve as important indicators of soil fertility [34,35]. The four soil enzymes selected in this study are key enzymes involved in the cycling of soil C, N, and P. ALP is an essential substance for microorganisms to increase the supply of available phosphorus under low-phosphorus stress [36]. BG primarily cleaves β-glucosidic bonds into utilizable glucose monomers, providing energy and carbon sources for microbial use [29]. In this study, the contents of soil total phosphorus and total carbon were lowest at moderate and high salinities. The activity of ALP was negatively correlated with the soil total phosphorus content, which was consistent with the ability of microorganisms to alleviate low-phosphorus stress by secreting ALP [34,36]. The activity of BG generally increased with increasing soil salinity, and its dynamics were notably coupled with the decomposition rate of lignins and the release of total carbon in the litter. These findings suggest that increasing salinity may accelerate the conversion of carbon in litter by enhancing the hydrolysis of β-glucosidic bonds [24]. This process is consistent with the net loss of soil total carbon at moderate and high salinities, indicating that these conditions may promote microorganisms to preferentially utilize readily decomposable carbon sources. LAP and NAG participate primarily in the decomposition of organic matter and the release of nitrogen. They can break down oligosaccharides such as chitin into monosaccharides, providing carbon and nitrogen sources that can be utilized by microorganisms, thereby promoting the degradation and circulation of soil organic matter [29,37]. The activity of LAP initially decreased and then increased with increasing soil salinity, whereas the activity of NAG gradually increased, peaking under high salinity. These trends were largely consistent with the changes in the soil water content and the contents of soil organic matter and total nitrogen. Additionally, the release of nutrients from the *T. chinensis* litter was greatest at moderate and high salinities. The findings of this study revealed that the soil enzyme activity in coastal saline–alkali soils in the Yellow River Delta tended to increase at slight, moderate and high salinities. These findings differ from the traditional understanding that salinity inhibits enzyme activity, as observed in a study on the effects of salinity stress on soil enzyme activity in the rhizosphere of *R. pseudoacacia* [38]. This phenomenon of stress-induced enhancement may be attributed to the experimental soil being sourced from coastal silt deposits of the Yellow River, which contain a significantly greater diversity and abundance of salt-tolerant microorganisms. These enzyme systems can maintain conformational stability at high osmotic pressure [5,10]. Additionally, the synergistic effect of humus and moisture may play a role in this process. Fulvic acid produced during litter decomposition can reduce ion toxicity by chelating Na^+^ [39], and an increased soil water content helps maintain the diffusion efficiency of enzyme-substrate interactions. These findings echo the very significant positive correlation between soil enzyme activities and microbial biomass in typical glacial watersheds on the Qinghai-Tibet Plateau [29]. When environmental stress does not exceed the tolerance threshold of microorganisms, biomass accumulation and increased enzyme activity can be achieved simultaneously. The degree of litter decomposition had no significant effect (*p* > 0.05) on the soil physicochemical properties or enzyme activities, which may be attributed to the short duration of this study and the gaps between the PVC isolation boards. In the future, long-term experiments will be conducted, and techniques such as metagenomics will be employed to analyze the regulatory networks of the expression of soil enzyme-encoding genes at different salinities. This will help reveal the molecular mechanisms underlying the response of the soil salinity–litter decomposition characteristics–enzyme activities chain.

## 4. Materials and Methods

### 4.1. Experimental Pool Setting in the Simulation

The experimental control area in the simulation is located at the experimental base of the Shandong Key Laboratory of Eco-Environmental Science for the Yellow River Delta, China. The area is within a warm–temperate semihumid continental monsoon climate zone. The annual average temperature is 12.6 °C, the annual average precipitation is 543.2 mm concentrated from June to September, the annual average frost-free period is 203 d, the annual sunshine duration is 2850 h, the annual average evaporation is 1806 mm, and the annual average wind speed is 3.1 m/s.

In terms of the total salinity content in agricultural soils, coastal saline–alkali soils are categorized mainly into nonsaline–alkali soils (<0.2%), slightly saline–alkali soils (0.2–0.4%), moderately saline–alkali soils (0.4–0.8%) and highly saline–alkali soils (1.0–1.5%) [40]. Therefore, four experimental pools with soil salinities of 0.1% (CK), 0.4% (SS), 0.8% (SM), and 1.2% (SH) were established for this simulated control experiment. Three replicates were established for each experimental pool, and a total of twelve experimental pools with a length of 1 m, width of 1 m, depth of 0.80 m, and volume of 0.80 m^3^ were constructed. Each experimental cell was closed at the bottom and around the perimeter to prevent salt loss, and a gap of 0.13 m was left at the top to facilitate the management of the application of water at a later stage. The experimental soil samples were taken from the silty soil of the coastal beach of the Yellow River and characterized as tidal soil formed by alluvial deposition in the area. The soil exhibits a fine and uniform texture with a loose structure and is classified as chalky loam (comprising 5.69% clay, 47.71% chalk and 46.60% sand), and the soil salt content is 0.1%. To ensure the randomness and reliability of the experiment, the locations of the experimental pools for different soil salinities were arranged randomly.

### 4.2. Collection and Treatment of T. chinensis Litter Samples

*T. chinensis* litter was collected as decomposition material from the *T. chinensis* forest in Binzhou Harbor, Binzhou city, Shandong Province, in the Yellow River Delta (118°3′15″–118°4′32″ E, 38°9′51″–38°10′44″ N). The litter was brought back to the laboratory to remove impurities and allowed to dry naturally to a constant weight. Decomposition bags were made from nylon mesh bags with dimensions of 25 cm × 15 cm and a mesh aperture of 0.5 mm. Undecomposed litter (NDL), semidecomposed litter (SDL), and already decomposed litter (ADL) were weighed into 15 g portions and placed into bags [41].

### 4.3. Experimental Setting

**Figure 8:**
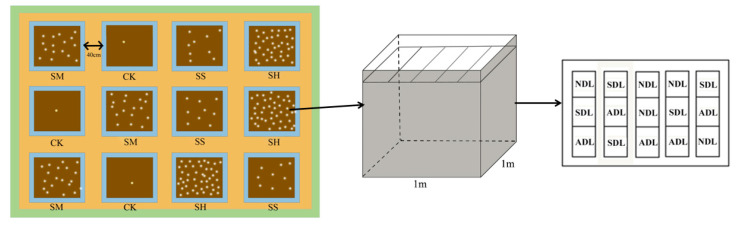
Diagram of the experimental layout (CK, SS, SM and SH analyses were repeated three times).

A simulated controlled experiment and the litterbag method were employed. Each experimental pool was divided into 15 equal plots, with 5 replicates each of NDL, SDL and ADL randomly placed within them. Each plot was isolated with 80 cm × 30 cm PVC boards to ensure that there was no mutual interference. One experimental pool was used as an example (Figure 8). *T. chinensis* litter with different degrees of decomposition was randomly placed in 12 experimental pools. The experimental design included 4 soil salinities, 3 replicates of experimental pools, 3 degrees of litter decomposition, and 5 replicates of samples, resulting in a total of 180 decomposition bags. The decomposition bags were laid out parallel to each other within the experimental pools without overlapping. The decomposition period was calculated starting from the date of the decomposition bag placement.

In this study, samples were collected in September 2023 after 180 d of litter decomposition. One bag of litter was collected for each of the three degrees of decomposition at the four soil salinities, with three replicates per treatment, resulting in a total of 36 bags; the corresponding soil samples were collected from the 0–10 cm layer. After they were returned to the laboratory, the soil and other debris were removed from the litter. The litter was then dried at 65 °C, crushed, and sieved through a 60-mesh screen for further analysis. For the soil samples, one portion was stored at 4 °C for the determination of soil enzyme activity levels, and the remaining portion was air dried, ground, and sieved through 20- and 100-mesh screens for further analysis.

### 4.4. Indicator Measurement

Litter chemical indicators: The contents of lignin, cellulose and hemicellulose were measured via the acid fiber washing method. The contents of total carbon and total nitrogen were determined using an elemental analyzer (Elementar, Vario EL III, Germany, Shanghai, China). The total phosphorus content was determined via the molybdenum–antimony–scandium colorimetric method.

Soil physicochemical indicators: The soil water content was measured via the drying method. The soil pH was measured with the potentiometric method. The content of soil organic matter was determined via the potassium dichromate oxidation method. The contents of total carbon and total nitrogen were determined using an elemental analyzer (Elementar, Vario EL III, Germany, Shanghai, China). The total phosphorus content was determined via the molybdenum–antimony–scandium colorimetric method. The activity of alkaline phosphatase (ALP) was determined via the disodium phenyl phosphate colorimetric method. The activity of β-glucosidase (BG) was determined via the salicin hydrolysis method. The activity of leucine aminopeptidase (LAP) was determined via the L-PAN method. The activity of N-acetylamino-glucosidase (NAG) was determined via the nitrophenol colorimetric method.

### 4.5. Data Processing

Microsoft Office Excel 2021 was used for data processing, and SPSS 27.0 was used for statistical analysis of the data. Two-way analysis of variance (ANOVA), one-way ANOVA and the Duncan method were used to test the significance of the differences between different treatments (*p* < 0.05). Spearman’s test was used to complete the correlation analysis. The lavaan and semPlot packages of R 4.3.2 were used for structural equation modeling (SEM) analysis and construction, and the ggplot2 package and PowerPoint of R Studio were used for drawing.

The specific code of the SEM method is as follows:

library(lavaan)
model < - ‘
# measurement model (latent and observed variables)
Latent1 = ~item1 + item2 + item3
Latent2 = ~item4 + item5 + item6
# structural model (relationship between latent variables)
Latent1~latent2

## 5. Conclusions

With increasing soil salinity, the residual substrate quality of the *T. chinensis* litter generally first decreased but then increased, reaching its lowest level under moderate salinity, for which the release of nutrients was maximized. The next-lowest quality was observed under high salinity. The physicochemical properties and enzyme activity of surface soil generally increased or first increased but then decreased, with the highest level observed under high salinity, followed by moderate salinity.

With increasing degrees of *T. chinensis* litter decomposition, the contents of lignin and cellulose residues in *T. chinensis* litter decreased, whereas the contents of hemicellulose, total nitrogen, and total phosphorus residues tended to increase. There was no significant change in the soil physicochemical properties or enzyme activities in response to the degree of *T. chinensis* litter decomposition.

SEM analysis indicated that soil salinity negatively influenced the residual substrate quality of *T. chinensis* litter and positively influenced the soil physicochemical properties, thereby influencing soil enzyme activities. The direct effects of the degree of *T. chinensis* litter decomposition on soil physicochemical properties were not significant.

In summary, soil salinity is not only a key driver of *T. chinensis* litter decomposition but also indirectly regulates enzyme activities by changing the soil microenvironment, thereby affecting soil nutrient cycling processes. The decomposition of the *T. chinensis* litter improved the enzyme activities of the surface soil to a greater extent in the moderate- and high-salinity-alkali coastal soils, whereas the effects of improvement were smaller in coastal slightly saline–alkali soils. However, owing to the short research time, limited research scope and experimental conditions, further exploration of the interactions between litter and soil and the corresponding mechanisms is necessary in the future to provide a scientific basis for improving soil quality in the coastal saline–alkali soils of the Yellow River Delta.

## Data Availability

Data are contained within the article.
